# Quantitative Drug-Susceptibility in Patients Treated for Multidrug-Resistant Tuberculosis in Bangladesh: Implications for Regimen Choice

**DOI:** 10.1371/journal.pone.0116795

**Published:** 2015-02-24

**Authors:** Scott K. Heysell, Shahriar Ahmed, Sara Sabrina Ferdous, Md. Siddiqur Rahman Khan, S. M. Mazidur Rahman, Jean Gratz, Md. Toufiq Rahman, Asif Mujtaba Mahmud, Eric R. Houpt, Sayera Banu

**Affiliations:** 1 Division of Infectious Diseases and International Health, University of Virginia, Charlottesville, Virginia, United States of America; 2 Mycobacteriology Laboratory, International Centre for Diarrhoeal Disease Research, Bangladesh (icddr,b), Dhaka, Bangladesh; 3 National Institute of Diseases of Chest and Hospital, Dhaka, Bangladesh; Fundació Institut d’Investigació en Ciències de la Salut Germans Trias i Pujol. Universitat Autònoma de Barcelona. CIBERES, SPAIN

## Abstract

**Background:**

Multidrug-resistant tuberculosis (MDR-TB) treatment in Bangladesh is empiric or based on qualitative drug-susceptibility testing (DST) by comparative growth in culture media with and without a single drug concentration.

**Methods:**

Adult patients were enrolled throughout Bangladesh during the period of 2011–2013 at MDR-TB treatment initiation. Quantitative DST by minimum inhibitory concentration (MIC) testing for 12 first and second-line anti-TB drugs was compared to pretreatment clinical characteristics and treatment outcomes. MIC values at or one dilution lower than the resistance breakpoint used for qualitative DST were categorized as borderline susceptible, and MIC values one or two dilutions greater as borderline resistant.

**Results:**

Seventy-four patients were enrolled with a mean age of 35 ±15 years, and 51 (69%) were men. Of the rifampin isolates with MIC >1.0 μg/ml, 12 (19%) were fully susceptible or borderline susceptible to rifabutin (MIC ≤0.5 μg/ml). Amikacin was fully susceptible in 73 isolates (99%), but kanamycin in only 54 (75%) (p<0.001). Ofloxacin was borderline susceptible in 64%, and fully susceptible in only 14 (19%) compared to 60 (81%) of isolates fully susceptible for moxifloxacin (p<0.001). Kanamycin non-susceptibility and receipt of the WHO Category IV regimen trended with interim treatment failure: adjusted odd ratios respectively of 5.4 [95% CI 0.82–36.2] (p = 0.08) and 7.2 [0.64–80.7] (p = 0.11).

**Conclusions:**

Quantitative MIC testing could impact MDR-TB regimen choice in Bangladesh. Comparative trials of higher dose or later generation fluoroquinolone, within class change from kanamycin to amikacin, and inclusion of rifabutin appear warranted.

## Introduction

Multidrug-resistant tuberculosis (MDR-TB) threatens to dismantle all prior gains in global TB control [1, 2]. Defined as resistance to rifampin and isoniazid, two key first-line medications, MDR-TB necessitates prolonged treatment with second-line medications of less efficacy and greater toxicity than those used to treat fully drug-susceptible TB [2]. Despite progress made in rapid molecular diagnosis for rifampin and isoniazid resistance, regimens of the very drugs used to treat MDR-TB in endemic areas are often empiric and designed without individual susceptibility testing, or based on limited qualitative methods performed at a national or supranational reference laboratory [3]. Such qualitative susceptibility testing is performed by comparative growth in culture media with and without a single drug concentration and does not provide the quantitative range of susceptibility present with minimum inhibitory concentration (MIC) testing using multiple dilutions.

Bangladesh is a World Health Organization (WHO) designated ‘high burden’ country for TB with estimates of MDR in 1.4% of new TB cases and 29% of previously treated cases [3]. An emerging majority of patients are diagnosed with MDR-TB following molecular testing of sputum by Xpert MTB/RIF (assay for *rpoB* gene mutation and rifampin resistance; Cepheid, CA, USA) or MTBDR*plus* (assay for *rpoB*, *inhA* and *katG* mutation predicting rifampin and low or high level isoniazid resistance respectively; Hain Lifescience, Nehren, Germany) [4, 5]. Patients referred to specialized hospitals for MDR-TB treatment are empirically started on one of two regimens depending on site-specific standards: 1) a standardized WHO regimen of five drugs (kanamycin given for at least 8 months; ofloxacin or levofloxacin; ethionamide or prothionamide; pyrazinamide; and cycloserine or para-aminosalicylic acid) given for at least 20 months, or if treated at one of the Damien Foundation supported centers, 2) the ‘Bangladesh’ regimen (gatifloxacin or moxifloxacin; clofazimine; ethambutol; pyrazinamide; and supplemented with kanamycin, higher dose isoniazid and prothionamide in the first 4 months) given for at least 9 months [6,7].

We hypothesized that the standardized approach to MDR-TB treatment may risk inclusion of medication to which an individual’s isolate is frankly resistant at high concentrations and medications near the borderline of resistance which may benefit from dose adjustment or in-class change, or the regimen may even exclude active medications for which full susceptibility is retained. We tested these hypotheses among subjects referred for MDR-TB treatment through a national drug-resistance surveillance project and for whom a pretreatment *Mycobacterium tuberculosis* isolate was available for MIC testing on the Sensititre MYCOTB plate (Trek Diagnostic Systems, OH, USA) [8]. Some *M*. *tuberculosis* isolates from this cohort were previously used to examine concordance of multiple methodologies of drug-susceptibility testing [9]. The MYCOTB MIC plate is a dry microdilution 96-well plate prefilled with lyophilized antibiotics representing 12 common first and second-line anti-TB medications that we and others have found compared favorably with conventional drug-susceptibility testing, and is now used as the primary phenotypic susceptibility platform at some large public health laboratories [9–13].

## Methods and Methods

### Setting

Patients were referred throughout Bangladesh during the period of 2011–2013 but primarily represented by the Dhaka, Chittagong, Rajshahi and Mymensingh regions. Adult patients were included as subjects if planned for initiation on a regimen for MDR-TB with an available pretreatment *M*. *tuberculosis* isolate, and excluded if second-line medications were used for other purposes, for example in the treatment of drug-susceptible TB with first-line drug intolerance. Demographics, co-morbidities, TB treatment exposure history, as well as symptom and disease severity of the current MDR-TB episode were collected by standardized interview and chart review. Attempt was made to contact patients after six and 12 months for repeat interviews. All patients signed informed consent and ethical approval for the study was obtained from the institutional review boards at the International Centre for Diarrheal Diseases Research, Bangladesh (icddr, b) and the University of Virginia.

### Laboratory procedures

All procedures were carried out at the Mycobacteriology Laboratory of the icddr, b in Dhaka by using pretreatment sputum specimens transferred from the referral sites. Sputum samples were digested and decontaminated by the NaOH method and cultured on Lowenstein Jensen (L-J) slants and in the automated Bactec MGIT 960 system (Becton, Dickinson, Franklin Lakes, NJ) [14,15]. Positive cultures were species confirmed by the Xpert MTB/RIF assay.

MIC testing on the 96-well Sensititre MYCOTB plate was performed as previously described [8]. MYCOTB plate results were performed in batch and not available for alteration of patient regimen. Briefly, suspensions of the cultured isolate were adjusted to 0.5 McFarland standard, and 100 μl of suspension was transferred into a tube containing 11 ml 7H9 broth supplemented with oleic acid-albumin-dextrose-catalase to yield 1x10^5^ CFU/ml. A 100 μl aliquot was transferred into each of the 96 wells, and the plate was covered with an adhesive seal and incubated at 37°C. Growth was evaluated visually with a manual viewer at 10 to 21 days by two independent technicians. The MIC was recorded as the lowest antibiotic concentration that reduced visible growth. The H37Rv laboratory strain was used for quality control.

MIC values for each drug were compared to the published single critical concentrations for the conventional agar proportion method [16]. As others have done, we first categorized an isolate as susceptible by MYCOTB if the MIC was less than or equal to the critical concentration in the agar proportion method, and resistant if the MIC was greater than the critical concentration [10]. Acknowledging that the MYCOTB plate does not include wells of identical concentrations to the agar proportion concentration for certain drugs (eg. ethambutol and cycloserine) and that MIC ranges on solid agar have previously been applied for clinical use at specialized centers [17, 18], we further chose to categorize MIC values at or one dilution lower than the critical concentration as borderline susceptible. MIC values one or two dilutions greater than the critical concentration were categorized as borderline resistant. The corresponding agar proportion critical concentrations and prefilled well concentrations on the MYCOTB plate for each drug are as follows for the first-line drugs: isoniazid MIC 0.25 μg/ml (pre-filled wells of 0.03, 0.06, 0.12, 0.25, 0.5, 1.0, 2.0 and 4.0 μg/ml); rifampin 1.0 μg/ml (0.12, 0.25, 0.5, 1.0, 2.0, 4.0, 8.0 and 16.0 μg/ml); rifabutin 0.5 μg/ml (0.12, 0.25, 0.5, 1.0, 2.0, 4.0, 8.0 and 16.0 μg/ml); ethambutol 5.0 μg/ml (0.5, 1.0, 2.0, 4.0, 8.0, 16.0 and 32.0 μg/ml); streptomycin 2.0 μg/ml (0.25, 0.5, 1.0, 2.0, 4.0, 8.0, 16.0 and 32.0 μg/ml); and the second-line drugs: kanamycin 5.0 μg/ml (0.6, 1.2, 2.5, 5.0, 10.0, 20.0, and 40.0 μg/ml); amikacin 4.0 μg/ml (0.12, 0.25, 0.5, 1.0, 2.0, 4.0, 8.0 and 16.0 μg/ml); ofloxacin 2.0 μg/ml (0.25, 0.5, 1.0, 2.0, 4.0, 8.0, 16.0 and 32.0 ìg/ml); moxifloxacin 2.0 μg/ml (0.06, 0.12, 0.25, 0.5, 1.0, 2.0, 4.0 and 8.0 μg/ml); ethionamide 5.0 μg /ml (0.3, 0.6, 1.2, 2.5, 5.0, 10.0, 20.0 and 40.0 μg /ml); cycloserine 25.0 μg/ml (2.0, 4.0, 8.0, 16.0, 32.0, 64.0, 128.0 and 256.0 μg/ml); para-aminosalicylic acid 2.0 μg /ml (0.5,1.0, 2.0, 4.0, 8.0, 16.0, 32.0 and 64.0 μg/ml). Quantitative susceptibility testing for clofazimine and pyrazinamide were not available.

### Statistical analysis

Data were entered into Microsoft Excel (Version 14.1.3) and analyzed using SPSS (Version 21). MIC distributions were reported as median values with intraquartile ranges (IQR), while the proportion of susceptible, borderline susceptible and resistant were reported as simple frequencies for each drug included on the MYCOTB plate. Comparison of susceptibility categories between two drugs within the same medication class, and clinical predictors of second-line drug susceptibility were made by chi-square or Fisher’s exact test when appropriate. Bivariate and multivariate logistic regression analyses were used to determine risk factors for interim treatment failure (death or failure to culture convert sputum when treated for pulmonary MDR-TB). All tests of significance were two-tailed.

## Results

Seventy-four patients met inclusion criteria with a mean age of 34 ±15 years. The majority, 51 (69%), were men and represented a diversity of occupations and potential TB exposures [Table 1]. Additional medical comorbidities were rare, or with respect to HIV and diabetes status, either not available or not assessed. Nearly all subjects, 73 (99%), reported a prior history of TB treatment. The majority of those with prior treatment had failed a first-line regimen but 10 (14%) had prior exposure to at least one second-line drug [Table 1]. Furthermore, 20 (27%) had completed treatment or been deemed as cured only to represent presumably with relapsed MDR-TB or re-infection with a new MDR strain. Most patients had a prolonged symptom duration and presented with body mass index (BMI) evidence of malnourishment, as 54 (73%) had a BMI of <18.5%.

**Table 1:** Demographics and pretreatment characteristics, N = 74.

Characteristic	Result
Age, mean years ±SD	34 ±15
Gender	
Male (%)	51 (69)
Female (%)	23 (31)
Occupation	
Business (%)	16 (21)
Domestic worker (%)	13 (18)
Agriculture (%)	6 (8)
Unemployed (%)	8 (11)
Garment worker (%)	6 (8)
Student (%)	6 (8)
Day labor (%)	6 (8)
Transportation (%)	5 (7)
Service (%)	5 (7)
Other (%)	3 (4)
History of incarceration	
Yes (%)	10 (14)
No (%)	64 (86)
Drug use	
Yes (%)	2 (3)
No (%)	71 (96)
Unknown (%)	1 (1)
Alcohol use	
Yes (%)	6 (8)
No (%)	68 (92)
Diabetes	
Yes (%)	7 (10)
No (%)	20 (27)
Unknown (%)	47 (63)
HIV infected	
Yes (%)	0
No (%)	62 (84)
Unknown (%)	12 (16)
Known TB contact	
Yes (%)	8 (11)
No (%)	66 (89)
Prior TB treatment	
Yes (%)	73 (99)
Unknown (%)	1 (1)
Result of most recent TB treatment (% of prior treatment)	
Failed first-line regimen	33 (45)
Failed regimen including second-line drugs	10 (14)
Cured or treatment complete	20 (28)
Defaulted	9 (12)
Other	1 (1)
Cough	
Yes (%)	69 (93)
Mean duration of cough in days ±SD	134 ±84
No (%)	5 (7)
Fever	
Yes (%)	49 (66)
Mean duration of fever in days ±SD	135 ±92
No (%)	25 (34)
Subjective weight loss	
Yes (%)	69 (93)
No (%)	5 (7)
Mean weight, kilograms ±SD	42.4 ±9.3
Mean body mass index (BMI) ±SD*	16.6 ±3.2
N with BMI<18.5% (% with BMI)	54 (73)

*Body mass index calculable in 72 patients.

### MIC distributions

Despite an alternative drug-susceptibility testing report of MDR-TB in the field prior to referral (eg. molecular test such as Xpert MTB/RIF) or phenotypic testing at another laboratory, 13% of isolates had a susceptible MIC for isoniazid, and 12% of tested isolates had a susceptible MIC for rifampin [Table 2]. Of the isolates susceptible or borderline susceptible to isoniazid, MTBDR*plus* results revealed *inhA* mutation only in 4, *katG* mutation only in 1, and the remaining 4 were wildtype for both regions based on repeat Genotype MDRTBplus testing. Similarly, of the isolates susceptible or borderline susceptible to rifampin, Xpert MTB/RIF testing revealed *rpoB* mutation in 4 (all with MIC of 1.0 μg/ml and including one isolate that was wildtype by MTBDR*plus*).

**Table 2:** Minimum inhibitory concentrations.

Medication	Median MIC μg/ml (IQR), [min; max]	Susceptible using APM critical concentration		Resistant using APM critical concentration	
		Susceptible (%N), [MIC range μg/ml]	Borderline susceptible (%N), [MIC range μg/ml]	Borderline resistant (%N), [MIC range μg/ml]	Resistant (%N), [MIC range μg/ml]
Isoniazid (N = 72)	4.0 (2.0–4.0), [0.06; >4.0]	2 (3), [<0.12]	7 (10), [0.12–0.25]	4 (5), [0.5–1.0]	59 (82), [>1.0]
Rifampin (N = 72)	>16.0 (>16.0),* [0.12; >16.0]	3 (4), [<0.5]	6 (8), [0.5–1.0]	2 (3), [2.0–4.0]	61 (85), [>4.0]
Rifabutin (N = 73)	4.0 (0.5–8.0), [≤0.12; >16.0]	7 (10), [<0.25]	14 (19), [0.25–0.5]	15 (20), [1.0–2.0]	37 (51), [>2.0]
Ethambutol (N = 72)	8.0 (4.0–8.0), [0.5; >32.0]	3 (4), [<2.0]	25 (35), [2.0–4.0]	40 (55), [8.0–16.0]	4 (6), [>16.0]
Streptomycin (N = 74)	4.0 (1.0–32.0), [0.25; >32.0]	14 (19), [<1.0]	20 (27), [1.0–2.0]	13 (18), [4.0–8.0]	27 (36), [>8.0]
Amikacin (N = 74)	0.25 (0.25–0.5), [0.12; 16.0]	73 (99), [<2.0]	0 [2.0–4.0]	1 (1), [8.0–16.0]	0 [>16.0]
Kanamycin (N = 74)	1.2 (1.2–2.5), [0.6; 40.0]	55 (74), [<2.5]	16 (22), [2.5–5.0]	2 (3), [10.0–20.0]	1 (1), [>20.0]
Ofloxacin (N = 74)	(1.0–2.0), [0.25; 16.0]	14 (19), [<1.0]	47 (64), [1.0–2.0]	4 (5), [4.0–8.0]	9 (12), [>8.0]
Moxifloxacin (N = 74)	0.5 (0.25–0.5), [0.06; 8.0]	60 (81), [<1.0]	5 (7), [1.0–2.0]	9 (12), [4.0–8.0]	0 [>8.0]
Ethionamide (N = 73)	2.5 (1.2–10.0), [0.6; >40.0]	21 (29), [<2.5]	26 (36), [2.5–5.0]	11 (15), [10.0–20.0]	15 (20), [>20.0]
PAS (N = 74)	(0.5–2.0), [≤0.5;>64.0]	26 (35), [<1.0]	39 (53), [1.0–2.0]	8 (11), [4.0–8.0]	1 (1), [>8.0]
Cycloserine (N = 74)	16.0 (16.0–32.0), [1.6; >256.0]	8 (11), [<8.0]	33 (45), [8.0–16.0]	32 (43), [32.0–64.0]	1 (1), [>64.0]

APM = agar proportion method. N for each drug indicates complete result and medications with any N<74 indicating an indeterminate MIC for that individual drug. PAS = para-aminosalicylic acid.

*No IQR reported as majority of isolates with MIC>16.0.

With regard to second-line MIC distribution, amikacin was found to retain full susceptibility in 73 isolates (99%), whereas kanamycin was fully susceptible in only 54 (75%)(p<0.001). The singular isolate with amikacin resistance (MIC 16.0 μg/ml) also had high-level kanamycin resistance (MIC 40.0 μg/ml). Similar in-class differences were observed for the fluoroquinolones where ofloxacin was borderline susceptible in 64%, and full susceptibility found in only 14 (19%) compared to 60 (81%) of isolates for moxifloxacin (p<0.001).

### Rifabutin susceptibility among rifampin resistant isolates

**Fig 1:**
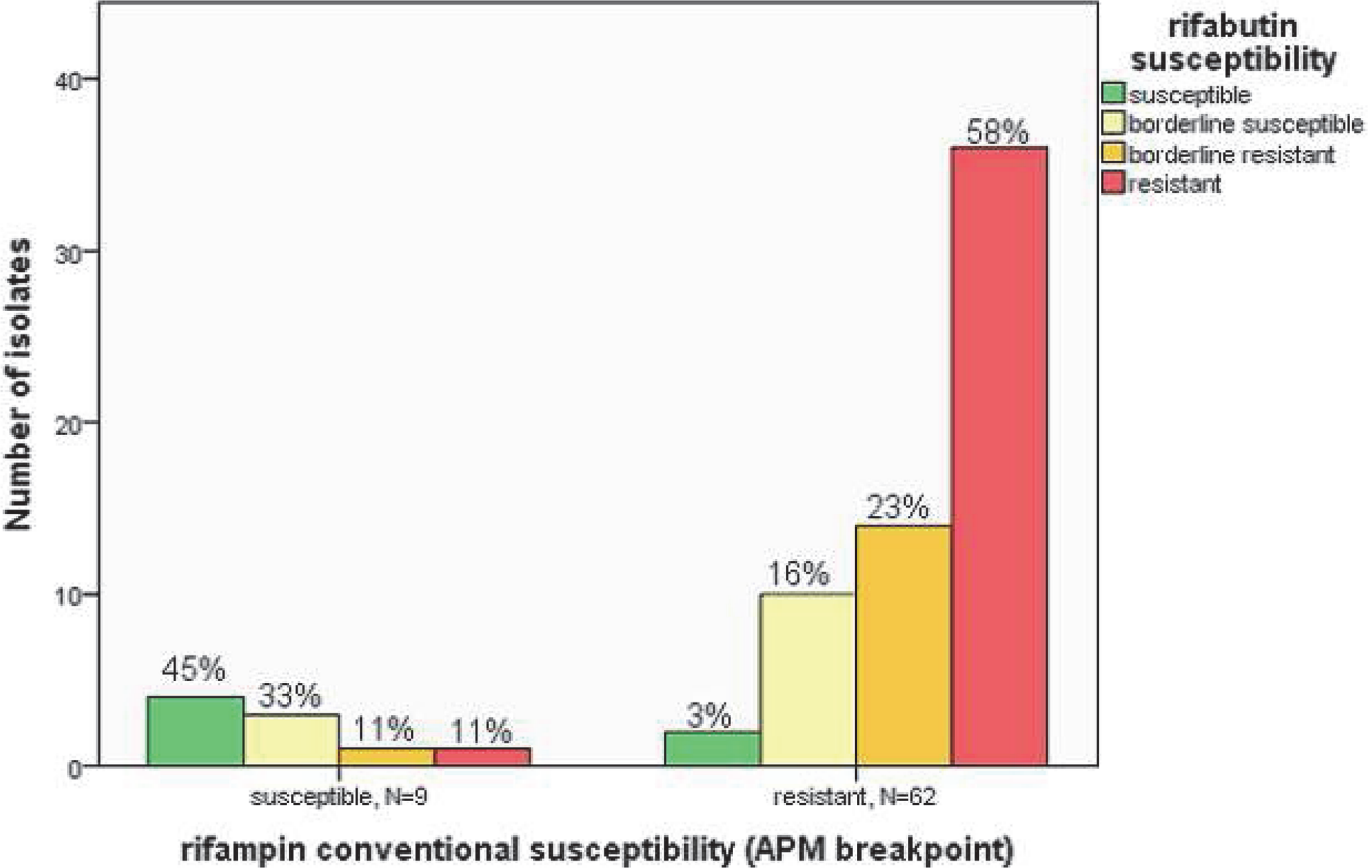
Rifabutin MIC distribution stratified among conventional rifampin-susceptible and rifampin-resistant isolates. Conventional rifampin susceptible by APM (agar proportion method) critical concentration (MIC≤1.0 μg/ml) and resistance (MIC >1.0 μg/ml). Rifabutin critical concentration = 0.5 μg/ml; susceptible (<0.25 μg/ml), borderline susceptible (0.25–0.5 μg/ml), borderline resistant (1.0–2.0 μg/ml) and resistant (>2.0 μg/ml). Percentages of N within each category of rifampin susceptible and resistant.

As rifabutin is a concentration dependent rifamycin of increasing interest in MDR-TB, we examined the MIC values of rifabutin among rifampin-susceptible and rifampin-resistant isolates, 71 isolates with complete MICs to both [Fig. 1]. Of the 62 isolates with rifampin resistance (rifampin MIC >1.0 μg/ml), 12 (19%) would have been considered susceptible to rifabutin using the conventional agar proportion breakpoint (rifabutin MIC ≤0.5 μg/ml). Yet another 14 (23%) were within the borderline resistant range. Deep sequencing of the *rpoB* region for these isolates was not performed.

### Clinical predictors of second-line drug susceptibility

Clinical predictors of susceptibility for key second-line drugs available in standardized regimens were stratified first by prior treatment success (treatment completion and/or microbiological cure) and failure (including default or other treatment interruption), but did not predict increased MIC [Table 3]. Similarly of the 73 subjects with a known outcome documented from the prior treatment episode, no trends were observed in relation to second-line drug susceptibility despite further refinement for those having specifically failed a prior regimen that included a second-line drug [Table 3].

**Table 3:** Predictors of second-line drug MIC increase, N = 73.

Medication	Prior treatment success (N = 20)	Prior treatment failure (N = 53)	P-value (chi-square)	No known SLD exposure (N = 63)	Failed with SLD regimen (N = 10)	P-value (chi-square)
Ofloxacin						
Susceptible	3 (15)	11 (21)	P = 0.74	13 (21)	1 (10)	P = 0.68
Non-susceptible	17 (85)	42 (79)		50 (79)	9 (90)	
Kanamycin						
Susceptible	14 (70)	41 (77)	P = 0.55	47 (75)	8 (80)	P>0.99
Non-susceptible	6 (30)	12 (23)		16 (25)	2 (20)	
Ethionamide*						
Susceptible	7 (37)	14 (26)	P = 0.40	20 (32)	1 (10)	P = 0.26
Non-susceptible	12 (63)	39 (74)		42 (68)	9 (90)	
Cycloserine						
Susceptible	5 (25)	8 (15)	P = 0.33	11 (18)	2 (20)	P>0.99
Non-susceptible	15 (75)	45 (85)		52 (82)	8 (80)	

SLD = second-line drug. Non-susceptible = MIC values of borderline susceptible, borderline resistant or resistant.

*Ethionamide MIC indeterminate in 1 isolate.

### Quantitative second-line drug susceptibility and treatment outcome

Interim treatment outcomes were available in 60 patients with pulmonary MDR-TB where the MDR regimen could be confirmed and the patient had not transferred, absconded or died from a non-TB related cause. Seven patients (12%) were categorized as having interim treatment failure (death or failure to culture convert sputum to negative at 12 months) including three deaths. As all MDR regimens included a fluoroquinolone, kanamycin and a thionamide (prothionamide or ethionamide), the MIC distributions of ofloxacin, kanamycin and ethionamide were studied as individual predictors of treatment failure, along with standard clinical characteristics and overall regimen choice [Table 4]. For each drug, a non-significant greater proportion of subjects with interim treatment failure had MICs in the non-susceptible range (borderline susceptible, borderline resistant or resistant). In adjusted regression analysis, kanamycin non-susceptibility and receipt of the WHO Category IV regimen continued to demonstrate a trend with treatment failure: adjusted odd ratios respectively of 5.4 [95% CI 0.82–36.2] (p = 0.08) and 7.2 [0.64–80.7] (p = 0.11), when including ofloxacin and ethionamide non-susceptibility in the multivariate model.

**Table 4:** MDR-TB treatment outcomes predicted by pretreatment characteristic, second-line medication susceptibility and overall regimen choice.

Characteristic	Living and culture conversion, N = 53	Death and/or no culture conversion, N = 7	Odd ratio [95% CI], p-value
Age			
<30 years	27 (51)	4 (57)	referent
30–49 years	18 (34)	3 (43)	1.1 [0.22–5.6], p = 0.89
≥50 years	8 (15)	0	n/c (p>0.99)
Gender			
Male	35 (66)	5 (71)	referent
Female	18 (34)	2 (29)	0.78 [0.14–4.4], p = 0.78
BMI <18.5%			
No	14 (28)	1 (14)	referent
Yes	37 (73)	6 (86)	2.3 [0.25–20.5], p = 0.47
Smoking			
No	36 (68)	4 (57)	referent
Yes	17 (32)	3 (43)	1.6 [0.32–7.9], p = 0.57
Diabetes			
No/Unknown	46 (87)	7 (100)	referent
Yes	7 (13)	0	n/c (p>0.99)
Ofloxacin			
Susceptible	12 (23)	1 (14)	referent
Non-susceptible	41 (77)	6 (86)	1.8 [0.19–16.0], p = 0.61
Kanamycin			
Susceptible	44 (83)	4 (57)	referent
Non-susceptible	9 (17)	3 (43)	3.7 [0.7–19.3], p = 0.13
Ethionamide			
Susceptible	16 (30)	1 (14)	referent
Non-susceptible	37 (70)	6 (86)	2.6 [0.29–23.3], p = 0.4
Regimen			
Bangladesh	22 (42)	1 (14)	referent
WHO (Cat IV)	31 (58)	6 (86)	4.26 [0.48–37.9], p = 0.19

n/c = not calculable. SLD = second-line drug. Non-susceptible = MIC value is borderline susceptible, borderline resistant or resistant. Culture conversion = sputum culture conversion to negative within 12 months of follow-up. WHO (Cat IV) = World Health Organization, category IV standardized regimen for MDR-TB; Bangladesh = ‘Bangladesh’ short-course regimen as detailed in Background.

## Discussion

Quantitative susceptibility testing of *M*. *tuberculosis* isolates in patients referred for treatment of MDR-TB in Bangladesh revealed important trends in MIC distribution including the majority of isolates with borderline susceptibility or resistance to ofloxacin, lower within class MICs for amikacin compared to kanamycin, and a surprisingly high number of isolates with fully susceptible or borderline susceptible MICs to the first-line medications isoniazid, rifampin and rifabutin. Rifabutin in particular, a first-line medication of limited availability for treatment of MDR-TB in many countries, was found non-resistant in 19% of all rifampin-resistant isolates, and to have MICs within two dilutions above the breakpoint for resistance in another 23%.

We and others have previously found discordance among common first-line drug-susceptibility tests that may be a function of true chromosomal mutation in resistance determining regions but which confer a low-level increase in MIC that is very near the single critical concentration employed for conventional phenotypic assays [9, 19–22]. Use of quantitative susceptibility testing as done with the commercially available MYCOTB plate can resolve this discrepancy and offers the clinician actionable data. For instance one may consider initiation of rifampin in the presence of a borderline susceptible MIC despite *rpoB* mutation, or higher dose rifampin with a borderline resistant MIC. We find this strategy of additional appeal given emerging evidence on the dose dependent bactericidal effect of rifampin and its safety and tolerability in therapeutic drug monitoring studies and high-dose clinical trials [23–25]. Certainly this is the rationale behind the initiation of high-dose isoniazid for isolates with only an *inhA* mutation [6, 7], but our findings demonstrate that for rare isolates even a *katG* mutation may be found with MIC values that retain borderline susceptibility. Practically, in settings where commercial molecular methods are used as the first test to begin a MDR-TB regimen, the MIC result would return 10–21 days after culture positivity hence employed for alteration and not initial construction of the multidrug-regimen.

Non-controlled study has suggested rifabutin-containing regimens used in the treatment of rifabutin-susceptible but rifampin-resistant MDR-TB were associated with higher treatment success compared to those with rifabutin-resistant MDR-TB that did not receive rifabutin despite similar drug-resistance patterns for other medications in the regimen [26]. Common mutations in *rpoB* at codons 526 and 531 are often found in *M*. *tuberculosis* isolates with high-level resistance to both rifampin and rifabutin [22, 27], while mutations at codons 516 and 522 have been associated with resistance to rifampin but susceptibility to rifabutin [22, 28]. More widespread use of rifabutin has been limited primarily by drug cost and availability of susceptibility testing [29]. While we acknowledge that susceptibility testing for rifabutin was performed exclusively by MIC testing in this study, given the increasingly understood economic burden of inadequately treating MDR-TB [30] and the reproducibility and ease of use of a platform like the MYCOTB plate [10], our findings support renewed examination of the best use of rifabutin in MDR-TB treatment.

Furthermore, the considerable proportion of isolates with borderline susceptibility to ofloxacin and kanamycin are of particular importance amidst the growing description of individual pharmacokinetic variability contributing to functional drug-resistance, whereby an isolate may be susceptible in vitro but inadequate circulating drug concentrations render a medication non-effective [31, 32]. Limited study of serum concentrations of MDR-TB drugs appear to correlate with tissue concentrations of lung specimens [33]. An illustrative example from MDR-TB patients treated in South Africa demonstrated ofloxacin target drug exposure, as measured by serum area under the concentration-time curve/MIC, was reached in only 45% of patients and in no patient with an ofloxacin MIC of 2.0 μg/ml, a MIC value within our categorization of borderline susceptible [34]. Our data support further study of fluoroquinolone dose optimization, as reports continue to suggest pharmacokinetic superiority of higher dose levofloxacin and the later generation moxifloxacin [35]. Indeed, MIC values near the conventional resistance breakpoint may in part explain the apparent benefit of continuing high-dose fluoroquinolone treatment in patients with fluoroquinolone-resistant MDR-TB [36]. In contrast, we harbor concern about the continued use of kanamycin as the injectable agent of choice, particularly given our prior findings of low peak concentrations of kanamycin in other MDR-TB settings [31] and the association of low-level kanamycin MIC increase with mutation in the *eis* promoter gene actually enhancing *M*. *tuberculosis* virulence [37, 38]. Compared to the fluoroquinolones, dose increase of the aminoglycosides may carry unavoidable toxicities. Thus in the absence of individualized serum drug concentration monitoring, amikacin appears the most effective empiric choice of this class given our available data (note capreomycin was not tested).

The trend in treatment outcome differences we observed highlights the necessity for rigorous prospective study to determine the impact of borderline susceptible MIC values within the context of individual pharmacokinetic variability and other important host factors. Primary limitations in the current analysis include the heterogeneity of result in the most recent TB episode prior to the patient’s presentation for MDR-TB treatment and the infrequency of certain potential clinical predictors such as second-line drug exposure, which may explain the lack of association with second-line drug MIC increase or the MDR-TB treatment outcome. Furthermore, for certain medications such as moxifloxacin susceptibility breakpoints are not well established on the MIC platform, or for medications such as cycloserine reproducibility may be poor in liquid media [38, 39]. Additional limitations include the lack of complete susceptibility data on all medications used in the MDR-TB regimen, notably pyrazinamide and clofazimine, which may have further obscured associations of outcome with quantitative susceptibility of the tested medications. Lastly, despite the apparent trend toward treatment success in patients that received the Bangladesh regimen, we could not control for other differences in clinical care that may have existed.

Nevertheless, we found quantitative susceptibility could significantly impact regimen choice, not the least of which may result in inclusion of first-line agents at normal or higher doses. As such, we propose the more clinically oriented use of ‘borderline’ susceptibility, particularly when categorizing medications at risk for frequently suboptimal circulating drug concentrations. Certainly more refined understanding of specific codon change within resistance determining regions of drug-resistance genes may ultimately predict quantitative change in MIC and additional study in this area is warranted. Yet in the absence of individualized MIC testing, more immediate local action could consider change of the injectable agent to amikacin from kanamycin, and selection of higher dose or newer generation fluoroquinolone as with the Bangladesh regimen. MDR-TB treatment trials that include rifabutin also appear most needed in this location, and we hypothesize such optimization could ultimately spare or significantly reduce exposure to the injectable agents.
